# Adult Neural Stem Cells: Basic Research and Production Strategies for Neurorestorative Therapy

**DOI:** 10.1155/2018/4835491

**Published:** 2018-04-01

**Authors:** E. M. Samoilova, V. A. Kalsin, N. M. Kushnir, D. A. Chistyakov, A. V. Troitskiy, V. P. Baklaushev

**Affiliations:** ^1^Federal Research Clinical Center of the Federal Biomedical Agency of Russian Federation, 28 Orekhovy Blvd, Moscow 115682, Russia; ^2^Department of Basic and Applied Neurobiology, V.P. Serbsky Federal Medical Research Center for Psychiatry and Narcology, Moscow, Russia; ^3^Institute for Advanced Studies, Federal Biomedical Agency of Russian Federation, Moscow, Russia

## Abstract

Over many decades, constructing genetically and phenotypically stable lines of neural stem cells (NSC) for clinical purposes with the aim of restoring irreversibly lost functions of nervous tissue has been one of the major goals for multiple research groups. The unique ability of stem cells to maintain their own pluripotent state even in the adult body has made them into the choice object of study. With the development of the technology for induced pluripotent stem cells (iPSCs) and direct transdifferentiation of somatic cells into the desired cell type, the initial research approaches based on the use of allogeneic NSCs from embryonic or fetal nervous tissue are gradually becoming a thing of the past. This review deals with basic molecular mechanisms for maintaining the pluripotent state of embryonic/induced stem and reprogrammed somatic cells, as well as with currently existing reprogramming strategies. The focus is on performing direct reprogramming while bypassing the stage of iPSCs which is known for genetic instability and an increased risk of tumorigenesis. A detailed description of various protocols for obtaining reprogrammed neural cells used in the therapy of the nervous system pathology is also provided.

## 1. Introduction

Initially, the technology of restoring pluripotency in differentiated cells was developed in 1952 by R. Briggs and T.J. King who used the method of nuclear transplantation [1]. However, the first truly pluripotent cells were the embryonic stem cells isolated in 1981 by two independent groups from early murine embryos [1]. At the same time, it was suggested that stimulation of proliferation and suppression of differentiation may be caused by certain factors presented in the cell medium [2]. This thought continued to develop actively gradually evolving into the hypothesis of directed cell reprogramming. Then, Hermann et al. [3] reported a paper comprising six protocols for the reprogramming of bone marrow-derived human mesenchymal stem cells (MSCs) into neural stem cells by adding a cocktail of factors to the cell medium. Of particular, various growth factors such as brain-derived neurotrophic factor (BDNF), platelet-derived growth factor (PDGF), epidermal growth factor (EGF), fibroblast growth factor 2 (FGF-2), and retinoic acid, a metabolite of vitamin A essential for support of cell growth, were used. The protocols of culturing cells both on a plane coated with poly-L-lysine and in neurospheres formed during cultivation on a low-adhesive coating were developed. At once, Takahashi and Yamanaka [4] reported the technique of generating pluripotent cells from differentiated somatic cells via retrovirus-mediated ectopic expression of four genes such as octamer-binding transcription factor 4 (Oct4), sex-determining region Y-box 2 (Sox2), Kruppel-like factor 4 (Klf4), and avian myelocytomatosis viral oncogene homolog (c-Myc). These four genes are now known as “Yamanaka factors” or OSKM factors. Beginning with this event, the era of genetic cell reprogramming without the use of nuclear transplantation has started.

In addition to the iPSC technology, the first reports about the possibility for direct transdifferentiation of somatic cells (including those of mesodermal origin) into neural stem/progenitor cells have appeared [5, 6]. In this review, we will focus on the key signal cascades involved in the acquisition and maintenance of pluripotency and also analyze the current protocols for obtaining neural stem cells from iPSCs as well as through direct transdifferentiation.

## 2. Pluripotency-Maintaining Mechanisms

Truly, pluripotent cells are embryonic stem cells (ESCs) derived from the internal cell mass in blastocysts. The pluripotency state of ESCs is characterized by the lack of cell differentiation and high proliferative activity. In ESCs, a biased cell cycle with a shortened G1-phase and an extended S-phase has been observed, which may be attributed to the reduced level of the cyclin-dependent kinases (CDKs) [7]. Increased telomerase activity is also typical for ESCs [8]. This is assumed to be due to the expression of the c-Myc transcription factor [9]. In addition, a number of transcription and chromatin remodeling factors is involved in maintaining the pluripotency of ESCs, that is, Oct4, Sox2, Klf4, Nanog [10, 11] as well as demethylases and histone deacetylases [12–16]. In adult stem cells, the same factors are involved in maintaining pluripotency. The formation and maintenance of pluripotency require activation or inhibition of multiple signaling pathways. Key signaling mechanisms are considered below.

### 2.1. Signaling Pathways

#### 2.1.1. Jak/Stat Signaling Pathway

This is a primary pathway for signaling from receptors of cytokines such as type I interferons, granulocyte colony-stimulating factor (G-CSF), and interleukins (IL-2, IL-3, IL-4, IL-6, IL-10, IL-12, and IL-13) [17]. In this signaling, a family of Janus protein kinases (JAK) and signal transducer and activator of transcription proteins (STATs) plays a role of key intracellular messengers. One of the most widely known factors triggering this cascade is the leukemia inhibitory factor (LIF), which belongs to the IL-6 family [18]. LIF effects appear to be mediated through a high-affinity receptor complex composed of a low-affinity LIF-binding chain (LIF receptor) and a high-affinity converter subunit, gp130. JAK is then activated and phosphorylates tyrosine amino acid residues in the receptor. The STAT transcription factor in turn can join them. The JAK kinase phosphorylates the STAT coupled with the receptor resulting in its dissociation from the receptor and further dimerization. The STAT dimer translocates into the nucleus and activates the expression of a network of genes including the genes that encode transcription factors Klf4 [1] and c-Myc [19].

#### 2.1.2. MAPK/ERK (MEK) Signaling Pathway

MEK is a prodifferential signaling pathway. It is activated in response to stimuli from tyrosine kinase receptors and receptors associated with G-proteins. A complex of molecules is assembled around the cytoplasmic domain of the activated receptor, which activates the Ras GTPase. It, in turn, sequentially activates Raf and MEK. These kinases activate the MAPK kinase, which through the phosphorylation of ERK regulates the expression of transcription factors, for example, by phosphorylating the transcription factor Nanog, thus reducing its transactivation [20]. Besides, as a result of ERK phosphorylation, c-Fos protooncogene transcription is activated that promotes the epithelial-mesenchymal transition and violation of the pluripotent status [21]. Therefore, on the one hand, inhibition of this pathway provides preservation of pluripotency [22]. On the other hand, this pathway upregulates the c-Myc gene activity and facilitates more active accumulation of cyclin D/Cdk4/6 complexes; thus, the complete knocking out of the Erk1 and Erk2 genes results in disruption of telomere function, premature launching the apoptosis system, and negatively affects the cell cycle [23–25].

#### 2.1.3. PI3K/Akt Signaling Pathway

PI3K/Akt pathway is closely related to the MAPK/ERK (MEK) signaling pathway [26], since it is indirectly activated via the effect of GTPase Ras on phosphatidylinositol-3-kinase (PI3K) [27]. This is a universal signaling pathway with a number of downstream cascades, one of which phosphorylates and activates protein kinase B, also known as Akt1 and plays a crucial role in maintaining pluripotency [28], primarily, due to suppression of apoptosis via inhibition of BAD proteins and caspase 9 [29, 30]. Besides, it indirectly provides a shift of the cell cycle from G1 to S-phase due to inhibition of GSK-3 protein kinase and the subsequent formation of the cyclin D/CDK complex [31]. Akt is also capable of direct regulation of inhibitors of cyclin-dependent kinase (CDK) p21 Cip1 and p27 Kip1, thus affecting the cell cycle [32]. Interestingly, while the MAPK/ERK pathway promotes PI3K activation via GTPase Ras, Akt blocks the MAPK/ERK pathway via inhibition of Raf protein kinase [33].

#### 2.1.4. Wnt/*β*-Catenin Signaling Pathway

The Wnt signaling pathway is necessary for a proper embryonic development and homeostasis of adult tissues. Among a number of descending cascading pathways, a canonical pathway is emphasized based on the stabilization of the cytoplasmic *β*-catenin protein. In the absence of a signal from the Wnt receptor, *β*-catenin is phosphorylated by a complex between adenomatous polyposis coli (APC), Axin-1, protein kinase GSK-3, and casein kinase (CK1). Binding of the ligand to the Wnt receptor initiates a cascade of events that inhibits this complex, which leads to the accumulation of *β*-catenin in the cytoplasm [34]. In case of excessive accumulation in the cytoplasm, *β*-catenin enters the nucleus where it activates transcription regulators like T-cell factor (TCF) and lymphoid enhancer-binding factor 1 (LEF1) and leads to the transcription of proproliferative genes such as c-Myc and cyclin D1 [35, 36]. In addition, *β*-catenin inactivates transcription factor 3 (Tcf3), which inhibits Oct3/4, Sox2, and Nanog and interacts with Oct4 thereby increasing its activity.

#### 2.1.5. Hedgehog (Hh) and Notch Signaling Pathways

The hedgehog (Hh) signaling pathway is involved in many developmental processes such as cell proliferation and differentiation including regulation of neural stem cells. In vertebrates, signal induction through this pathway is represented by three main ligands: sonic hedgehog (SHH), Indian hedgehog (IHH) (both are involved in the proneural development), and desert hedgehog (DHH) [37]. The signal transduction via the Hh pathway is mediated by the interaction between PTC1, a protein phosphatase and smoothened (SMO) proteins. It is assumed that PTC1 acts through a catalytic mechanism, and its absence adversely affects SMO activity [38]. In the inactive state, PTC1 represses SMO, and an inactive complex is formed in the cytoplasm with assembly with Costal 2 (Cos2), Fused (Fu), suppressor of Fu (SuFu), and Cubitus interruptus (Ci), all associated with microtubules. The stability of the complex is mediated by protein kinase A (PKA), casein kinase I*α* (CKI*α*), and glycogen synthase kinase 3*β* (Gsk3*β*). PTC1-mediated phosphorylation leads to the downregulation of SMO, so that SMO is activated by phosphorylation. This facilitates the sequential activation of Cos2 and Fu followed by the release Ci from the complex and subsequent activation. Next, Ci translocates to the nucleus, where it acts as a transcription factor [39]. This pathway is most strongly involved in the embryonic development. In the adult organism, this pathway is usually not activated except for tissue regeneration-triggering cases.

The Notch signaling pathway is an important regulator of intercellular interaction during embryogenesis, cell proliferation, differentiation, and apoptosis [39]. The Notch pathway is a relatively short; it includes one of the four membrane receptors Notch (1–4), the extracellular part of which when bound to a ligand undergoes proteolytic cleavage with an ADAM-type metalloprotease, and its intracellular part is cleaved by *γ*-secretase. The intracellular portion of the Notch protein remaining after cleavage translocates to the nucleus where it forms a transcriptional complex with the RBPJ protein (CBF1) and acts as a transcription factor [40]. Activation of this pathway is necessary to maintain the proliferation and pluripotency of human ESCs and the normal functioning of adult cells.

#### 2.1.6. TGF-*β* Signaling Pathway

The TGF-*β* signaling pathway plays an important role in development. TGF-*β* can induce apoptosis by activating one of the two signaling pathways such as SMAD or DAXX. TGF-*β* dimer binds to a second type receptor (TGFBR2) that is then assembled with the first type receptor (TGFBR1) followed by its phosphorylation. Further, signaling events involve interacting and phosphorylating the R-SMAD receptor and SMAD3. SMAD3 forms a heterodimeric complex with SMAD4 that enters the nucleus and acts as a transcription factor for various genes including proapoptotic genes [41]. The SMAD pathway is subdivided into Smad1/5/8- and Smad2/3-dependent branches. The Smad1/5/8 branch involves bone morphogenetic proteins (BMPs), in particular, BMP4, and is believed to play an important role in maintaining the activity of the LIF-STAT3 pathway [42]. The Smad1/5/8 pathway is inhibited by the upregulation of the Smad2/3 pathway, which conducts signals from activin A that stimulates the expression of pluripotency factors such as Nanog, Oct4, FGF-2/8, and NODAL. Also, Smad3 independently forms a complex with Oct4 and directly regulates many of its targets [43]. TGF-*β* also triggers apoptosis with DAXX (death-associated protein 6), predominantly in lymphocytes and hepatocytes.

#### 2.1.7. FGF Signaling Pathway

Most proteins of the fibroblast growth factor family are autophosphorylated through the ligand-receptor interaction and transmit the signal via the four main pathways: RAS-RAF-MARK (ERK), PLC*γ*-PKC, PI3K-AKT, and JAK/STAT, which in turn lead to the activation of various transcription factors. For example, FGF2, the main fibroblast growth factor, through the Smad2/3 pathway together with activin A, stimulates the expression of Nanog [44].

Obviously, none of the abovementioned signaling pathways is capable of independently maintaining the state of pluripotency; however, a complex effect on them may allow, although not completely, to control the proliferation, differentiation, and development of cells. As mentioned above, these signaling mechanisms typically lead to the activation of a variety of transcription factors including Oct3/4, Sox2, Nanog, Klf4, and c-Myc. In addition, some chromatin modulators, matrix, and microRNAs (miRs) contribute to the maintenance of pluripotency, as well as, in a case of experimental reprogramming of somatic cells, recombinant proteins, and small molecules do.

### 2.2. Transcription Factors

Transcription factors Oct3/4 (Pou5f1), Sox2, and Nanog are considered to be the main ones that form pluripotency. In addition to these, there are additional factors, such as Sall4, Klf4, and Stat3 as well as an important multiprotein complex represented by the Myc oncoprotein.

#### 2.2.1. Oct4

Oct4 belongs to the POU family of homeodomain proteins and is encoded by the Pou5f1 gene. Initially, its expression was determined in ESCs and carcinoma cells [45]. Studies have shown that the inhibition of Oct4 in murine ESCs contributed to their differentiation into trophectoderm cells while increased expression of Oct4 caused transformation into the primitive endoderm and mesoderm, which may be possibly due to Oct4-mediated downregulation of the CDX2 expression [46]. Some Oct4 targets are known including FGF4, Utf1, OPN, Rex1/Zfp42, Fbx15, and Sox2, and all are pluripotent transcription factors [47–49]. Oct4 collaborates with those factors to regulate the expression level of pluripotency genes for example such as Nanog. However, upregulated Sox17 may replace Sox2 as a partner for Oct4 and cause a switch from a pluripotency to endodermal differentiation [50]. Oct4 may regulate expression of target genes both as a heterodimer with Sox2 [47] as well as in monomeric and homodimeric forms, for example, in the case of OPN [51].

#### 2.2.2. Sox2

Sox2 (SRY- (sex-determining region Y-) box 2) belongs to the SOXB1 subgroup within a family of transcription factors with a single high mobility DNA-binding domain [52]. Sox2 is expressed in adult cells and in the internal cell mass of blastocysts [53]. A part of the Sox2 target genes overlaps with the Oct4 target genes, in particular, genes such as Fgf4, Utf1, Fbx15, and Nanog [47, 54–56]. Sox2-deficient murine ESCs differentiate into trophectoderm but increased expression of Oct4 supports cell pluripotency even in the knockout of the Sox2 gene, apparently due to synergistic expression of Sox2 and Oct4. However, overexpression of Sox2 in the ESCs may negatively affect the maintenance of their proliferation [57], but the mechanism of this effect is still not clear.

Sox2 shares an expression network with Oct4, and after forming a complex, it regulates the work of target genes. Initially, Sox2 binds to the promoter or enhancer sequences of target genes, and heteromerization with Oct4 stabilizes this binding [58]. The Oct4/Sox2 complex interacts with the target DNA sequences in a double fashion. The complex can bind to consecutive “canonical” sites without breaks and also join the motifs separated by several pairs of bases [59].

#### 2.2.3. Nanog

Nanog is a homeodomain transcription factor involved in the self-renewal of undifferentiated ESC. It acts as an activator of LIF [60], but when overexpressed, this factor is able to maintain cell proliferation in the absence of LIF [61]. Interestingly, Nanog-deficient murine ESCs, although prone to differentiation, can still retain pluripotency [61]. Nanog was suggested to regulate proliferation both by inhibiting the expression of certain genes, for example, Gata4 and Gata6, which promote differentiation into a primitive endoderm [61], as well as through the activation of others such as Rex1 [62]. Moreover, Rex1 is also a target gene for the Oct4/Sox2 complex.

As a target for Oct4 and Sox2, Nanog in turn may reciprocally stimulate the synergistic activity of this pair, in particular, by activating the promoter of the Oct4 gene [63]. In fact, these three factors form a regulatory network that dynamically supports cell proliferation [64]. In the promoter of the Nanog gene, there is a binding site for the Oct4/Sox2 heterodimeric complex [65]. Oct4/Sox2 stimulates Nanog expression, but when Nanog is overproduced the heterodimer inhibits its expression [63]. The mutual influence of these three factors is a part of a large-scale network responsible for maintaining pluripotency. Their preferential targets are genes encoding STAT3, ZIC3, and also Oct4, Sox2, and Nanog themselves. This indicates an autoregulatory pattern of the activity control for these factors, as well as the genes of signaling pathway components. All three factors together upregulate teratocarcinoma-derived growth factor 1 (TDGF1), left-right determination factor 2 (LEFTY2), dickkopf-related protein 1 (DKK1), and GSK-3-binding protein. TDGF1 and LEFTY2 are the components of the TGF-*β* signaling while DKK1 and FRAT2 are involved in the Wnt signaling mechanism [66]. In addition to the positive regulation of those genes that are important to maintain the proliferation, Oct4, Sox2, and Nanog inhibit the activity of a number of prodifferentiation transcription factors active in the embryonic period, for example, ESX1I, HOXB1, MEIS1, LHX5, LBX1, MYF5, and ONECUT1 [66]. Oct4, Sox2, and Nanog also regulate expression of some microRNA (miRs) including miR-296, miR-302, miR-137, and miR-124a, which are important for the normal development of the nerve tissue [66, 67].

Propluripotent transcription factors are able to directly interact with each other forming protein complexes. For example, Nanog can bind to a SMAD1 and then suppresses bone morphogenetic protein 4- (BMP4-) mediated differentiation pathway [68]. Nanog also interacts with transcriptional regulators such as Oct4, DAX1, NAC1, ZFP281, and SALL4 [69], thereby forming through them a link with a number of other regulatory proteins, including the proteins of the Polycomb group [69]. Oct4 shares some common partner proteins with Nanog, for example, SALL4, ZFP281, and DAX1. In addition, Oct4 interacts with the transcription factors such as KLF4, SOX2, and TCFCP2L1 and forms complexes with the components of epigenetic machinery such as NuRD, SWI/SNF, PRC1, MYST2, and DNMT3A [70, 71]. Thus, the main factors of maintaining pluripotency are characterized by the complexity of interaction.

#### 2.2.4. Klf4

Klf4 belongs to the family of Kruppel-like factors, which are “zinc fingers” transcription factors involved in the control of expression of both proliferative and differentiative genes. Thus, the use of RNA interference (RNAi) to inhibit Klf4 induces differentiation of ESCs [72] while increased expression of Klf4 stops differentiation and enhances Oct4 expression [73]. Also, Klf4 in combination with Oct4 and Sox2 enhances the expression of Nanog [74]. As mentioned above, Klf4 is directly targeted by the STAT3 signaling pathway and Nanog [75]. However, Klf4 increases levels of a negative cell cycle regulator p21 [65], which increases differentiation but also downregulates p53, a proapoptotic regulator [76]. Simultaneously, Klf4 cooperates with Oct4 and Sox2 in the suppression of the p21-encoding Ink4 locus, which then promotes the maintenance of proliferation [77].

#### 2.2.5. Myc

The Myc family, which includes c-Myc, N-Myc, and L-Myc, is unique since its members act not only as classical transcription factors but also able to affect the structure of chromatin by activating histone acetyltransferases [78]. Myc is activated through many signaling pathways including LIF/STAT3, Wnt, Shh, and MAPK/ERK. Myc plays a key role in proliferation, differentiation, and apoptosis. As a typical prooncogene, it provides an option for cells to maintain pluripotency. Interestingly, although separate inactivation of c-Myc and N-Myc does not have any effect on pluripotency, their concomitant suppression leads to differentiation into primitive endoderm and mesoderm [79]. However, it was shown that Myc suppresses the MAPK signaling mechanism and, as a result, inhibits differentiation [80]. The shifted G1-S transition of the cell cycle is a characteristic of the pluripotency that promotes the proliferation rate. This may be attributed to the control of activity of CDKs [81], probably due to the activity of Myc proteins including c-Myc in response to STAT-dependent pathway activation [19]. Klf4 and c-Myc alone are sufficient to induce cell reprogramming, but the combination with at least one of the pluripotent factors such as Oct4, Sox2, and Nanog may increase the efficiency of reprogramming [82].

In summary, it should be stressed that Oct4, Nanog, Sox2, Klf4, and Myc play a crucial role in the maintenance of pluripotency of cells. All these factors are also strong prooncogenes since they stimulate proliferation and block differentiation. Normally, these factors are critically involved in the control of embryogenesis. However, overexpression of pluripotency transcription factors may increase the risk of malignization and oncogenicity, a fact that should be taken into account when iPSC technology is clinically implemented.

### 2.3. Epigenetic Factors

#### 2.3.1. MicroRNAs

MiRs are small noncoding RNAs that are involved in epigenetic regulation of the cell cycle, apoptosis, proliferation, migration, and differentiation [83]. Mutations in the Dicer and Dgcr8 genes, both are key elements of the MiR biogenesis, lead to the disruption of cell cycle and loss of the control of proliferation and differentiation [84, 85]. Certain miRs regulate and maintain the pluripotent state of the ESCs. For example, miR-302 and miR-290 regulate expression of Oct4, Sox2, and Nanog; these miRs are not detected in differentiated cells [86–88]. By contrary, another population of miRs may be found only in the adult somatic cells, in which they maintain a differentiated state [84]. For example, miR-134 performs this via inhibition of Nanog [89]. The function of Oct4, Sox2, Nanog, and miRs is reciprocally interrelated. For instance, miR-134, miR-296, and miR-470 can suppress the activity of Oct4, Nanog, and Sox2 [90]. miR-200c, miR-203, and miR-183 inhibit Sox2 and Klf4 [91]. On the other hand, Lin28, a transcription regulator, downregulates expression of the let7 miR family, a robust tumor suppressor that triggers cell differentiation [92]. At present, researchers who develop artificial pluripotent cells use miR transfection as an alternative to traditional transcription reprogramming factors, in particular miR-200c, miR-291-3p, miR-294, miR-295 [93], miR-200c, miR-302s, miR-369s [94], miR-302, and miR-367 [95].

#### 2.3.2. Chromatin Modifications

Epigenetic modifications of chromatin play a key role in the regulation of gene expression. Epigenetic mechanisms influence the accessibility of transcription regulators to gene promoters and enhancers, including genes involved in maintaining the pluripotency state. Chromatin modifications include DNA methylation/demethylation, histone modifications, and topological changes in the structure of chromatin.

DNA methylation is a reversible epigenetic modification that is mediated by DNA methyltransferases, a family of enzymes that catalyzes methylation of cytosine to form 5-methylcytosine (5mC). In mammals, the most studied members of the DNA methyltransferase family (DNMT) are DNMT1, DNMT3a, and DNMT3b. DNMT1 is responsible for replicative methylation of DNA, while DNMT3a and DNMT3b are involved in de novo methylation [96]. In totipotent zygotes, chromosomal DNA is almost unmethylated, but the methylation rate gradually increases during differentiation [97, 98]. Compared with totipotent zygotes, the genome of ESCs is methylated [99], which obviously indicates the transition from the totipotency to pluripotency. However, in terminally differentiated cells, the level of genome methylation is sharply reduced [98]. The more differentiated the cells become, the stronger the promoter regions of the Oct4 and Nanog are methylated in those cells [99].

Methylated centromeres and precentromeric regions of DNA are important for the correct formation of heterochromatin. CpG islets located in the regulatory regions such as promoters or enhancers are a preferential target for methylation [100]. Recently, a TET methylcytosine dioxygenase family enzymes have been found. TET enzymes catalyze the oxidation of 5-methylcytosine (5mC) to 5-hydroxymethylcytosine (5hmC), so they can act as demethylases by performing oxidative demethylation. The mammalian TET family consists of three members: TET1, TET2, and TET3. ESCs are highly expressed TET1 and TET2 that are necessary for demethylation of the genes responsible for pluripotency. Expression of TET1 and TET2 decreases during differentiation. TET3 is induced in germ cells at the onset of embryogenesis [101]. Usually, 5mC is localized in the centromeric and precentromeric regions of chromosomes while 5hmC is located in CpG islets of the chromosomal shoulders [102, 103].

Generally, chromatin modifications involve DNA methylation and posttranslational modifications of histones [104]. The latter include histone acetylation, methylation, phosphorylation, ubiquitination, summation, biotinylation, and citrullination. We just mention only some of them. Enzymatic histone modifiers include a variety of factors such as histone methyltransferases (HMTs), the proteins of the Polycomb group, demethylases (HDMs), histone acetyltransferases (HATs), and histone deacetylases (HDACs). Histone 3 (H3) is a typical target to be epigenetically modified. The most common modifications are the methylation of lysine 4 (H3K4me), lysine 9 (H3K9me), lysine 27 (H3K27me), and lysine 36 (H3K36me3), as well as acetylation of lysine 27 (H3K27ac). Modifications of H3K4me3, H3K9ac, H3K27ac, and H3K36me3 can be preferentially met in the euchromatic regions while H3K9me3 and H3K27me3 are predominantly distributed in the heterochromatic regions [105].

Members of the Polycomb group (PCG), which have the histone methyltransferase activity, are crucially involved in chromatin remodeling and epigenetic silencing of genes. There are two PGS-related protein complexes such as Polycomb repressive complex 1 (PRC1) and PRC2. Generally, the complexes catalyze the formation of repressive epigenetic marks at histone 3 such as H3K27 and H3K9, a hallmark of heterochromatic regions, and the permissive H3K4 mark, which is associated with the formation of euchromatin [104]. PCG proteins are involved in the regulation of expression of Oct4, Sox2, and Nanog [106]. In the modified histones, there are bivalent domains which may act as suppressors in the presence of the H3K27 or as activators in the presence of H3K4 [100]. These genome areas are commonly associated with a poor expression of transcription factors.

The activity of transcription factors and the state of chromatin are tightly associated. On the other hand, not only the local chromatin architecture affects the activity of genes but also the transcription factors can reciprocally trigger chromatin modifications. For example, Oct4, Sox2, and Nanog jointly regulate expression of genes encoding histone modifiers such as SMARCAD1, MYS3, and SET [66]. In addition, they also directly or indirectly interact with the histone-modifying enzymes such as histone deacetylases NuRD and PCG and therefore activate gene expression [69].

The balance between heterochromatin and euchromatin is very dynamic. Generally, in pluripotent cells, the balance is shifted towards the euchromatin [107]. Pluripotent stem cells have a unique higher-order genome structure shaped by pluripotency factors [108].

As will be further shown, small molecules can induce a “nonclassical” reprogramming, with the release of factors that are able to initiate the modification of chromatin.

#### 2.3.3. Small Molecules

**Figure 1:**
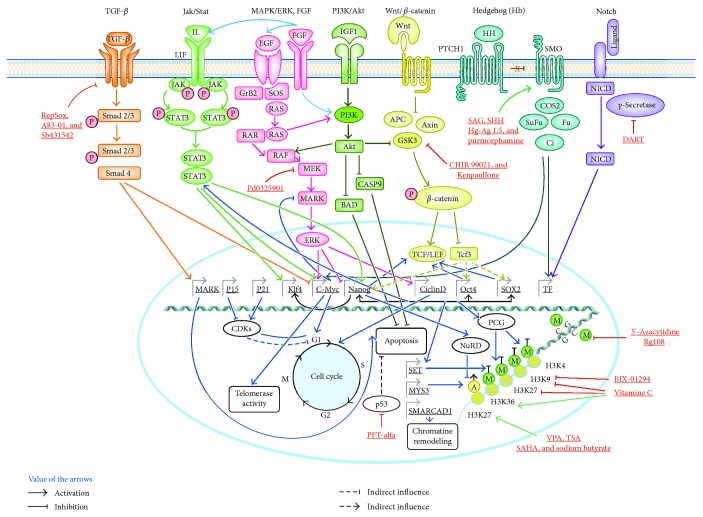
A diagram of signal pathways and molecules participating in the preservation of the pluripotency state and reprogramming. Modulation of some signal pathways provides preservation/return of the pluripotent state of the cell. Activation of the Notch, JAK/STAT, PI3K/Akt, Wnt, and hedgehog signal pathways upregulates expression of the pluripotency genes, the genes which shift the cell cycle towards S-phase and directly or indirectly block the apoptosis system, while complete or incomplete inhibition of the TGF-b and MAPK/ERK signal pathways removes the block from transcription of some transcription factors of pluripotency and prevents activation of the apoptosis system. In the diagram, each signal pathway and all its effects are labeled with an individual color, activating and inhibiting effects are marked. Cyan arrows point the signal conduction from FGFs via different signal pathways. Black arrows point a synergic effect of transcription factors on each other. Blue arrows show the effect of transcription factors on other genes and systems (e.g., on the apoptosis system) and interaction with protein complexes (e.g., proteins of the Polycomb system and NuRD). Small molecules are marked with the red font. Green arrows show the activating effect of small molecules, and red arrows show the inhibiting effect of the small molecules. P: phosphorylation; М: methylation; А: acetylation.

The epigenetic reprogramming may be performed with help of small chemical drugs. The technology provides many advantages like an option of monitoring of all stages of reprogramming, reversibility, low-cost, and a relatively high efficiency. Small molecules applied for reprogramming involve epigenetic modifiers, signal modulators, metabolic regulators, controllers of cell apoptosis, and substances that affect the state of chromatin and histones (Figure 1).

Small chemical drugs include various low-molecular-weight compounds that may influence reprogramming efficiency, with prominent examples such as valproic acid (an inhibitor of HDACs), azacytidine and RG108 (both are inhibitors of DNA methylation), and forskolin (an activator of adenylyl cyclase) [109, 110]. Other small molecules may indirectly influence the pluripotency by affecting the expression/activity of pluripotent factors such as purmorphamine (a SHH agonist), CHIR99021 (a GSK3*β* inhibitor), and A-83-01 (a TGF-*β* inhibitor) [111, 112]. Of note, GSK3*β* and TGF-*β* pathway inhibitors were shown to be successfully used to induce NPCs [113–115].

Interestingly, some studies have shown the potential of small molecules as a substitute for OSKM factors by reprogramming in NSCs, although most researchers prefer to combine transcription factors with small molecules to increase the efficiency of obtaining neural clones from somatic cells [114]. Separately, the role of small molecules as epigenetic regulators that modulate the processes of DNA methylation and chromatin modification should be emphasized. For example, chemical analogs of TET enzymes are isolated, such as the methyltransferase DNA inhibitor 5′-azacitidine and RG108 [114]. Also, among the small molecules, there are several substances acting as inhibitors of histone deacetylases: suberoylanilide hydroxamic acid (SAHA), trichostatin A (TSA), and valproic acid (VPA) [114]. A BIX01294 is an inhibitor of G9a histone methyltransferases that methylate histones of H3 in the position of lysine 9 (H3K9) [115] (Figure 1).

## 3. Approaches for Delivery of Pluripotent Factors into the Cell

### 3.1. Genetic Modification

Delivery of reprogramming factors to stem cells may stimulate the performance of reprogramming. In first experiments of generating iPSCs, a cocktail of Oct3/4, Sox2, Klf4, c-Myc, (classically called “OSKM”) factors were delivered to mouse fibroblasts by transfection of Moloney murine leukemia virus (MMLV) [4]. Subsequently, a retroviral transduction was efficiently used for reprogramming murine and human fibroblasts, neural stem cells, keratinocytes, adipocytes, and other cells. The efficiency of reprogramming by this approach has been relatively low, although higher than when using plasmid vectors [116]. In addition, retroviral transduction was completely ineffective for nondividing cells such as neurons. Lentiviral vectors showed a more potency to deliver genetic constructs to slowly dividing cells such as neurons [117]. The major disadvantage of viral-associated delivery of factors to the cell genome is the emergence of genomic instability, mutagenesis, and the probability of malignant transformation of cells due to the integration of large virus structures into the genome including the protumorigenic nature of delivered transcription factors.

To escape this pitfall, integration-defective retroviruses that are potentially less harmful were constructed. That excited a great interest, but the extremely low efficiency of this technology did not provide a good hope of the construction of clinically effective iPSCs [118].

Alternately to viral transduction, the approach of a polycistronic cassettes delivery of OSKM factors with transposons like PiggyBac (PB), a mobile genetic element, was also explored [119]. The PB transposase recognizes sequences of inverse terminal repeats (ITRs) of about 13 bp at both ends of the transposon and integrates those into sequences of the TTAA site. The efficiency of this technology is comparable to the retroviral approach. However, it is impossible to completely remove transposons after a procedure because of multiple copies [117]. Other transposons were used for reprogramming such as Sleeping Beauty (SB), whose efficiency is higher than that of PB, but its subsequent removal from the cell also causes great difficulties, which can also be dangerous in terms of destabilizing the genome [120].

Another method is the use of replicatively incompetent adenoviral vectors expressing OSKM factors [121]. Despite the fact that these vectors do not integrate into the genome of the cell, expression can last for several days. Although the safety of such vectors is significantly higher than that of retroviral vectors, the efficiency of their transduction is much lower [121]. As a nonviral vector, a miniring DNA containing genes of transcription factors were implemented [122]. Though this method also does not involve integration into the genome, its effectiveness is significantly lower than the retroviral technique [123].

In addition to integrating and nonintegrating vectors, a method that uses single-stranded RNA transduction by Sendai virus (SV) was developed [124]. SV is not pathogenic to humans, since it does not integrate into the genome and can be easily removed by a negative selection of antibodies. In addition, the efficiency of this method is comparable to that of a lentiviral transduction. However, this method also has its own limitations since viral replicates are very vulnerable to the nature of the transferred factors. As an alternative to vectors, some researchers use *in vitro* electroporation, but its effectiveness is rather low [95].

### 3.2. The Use of mRNA and Recombinant Proteins

The idea of using mRNA as an alternative to direct genetic effects in reprogramming is promising for two resons: it is safer, since the iPSCs are being constructed with mRNA (as well as directly reprogrammed cells), and it shows reduced immunogenicity of the resulting cells. But despite the safety of the method and its rather high efficiency, it remains extremely expensive and time-consuming [125].

For cell reprogramming, purified OSKM transcription factors can be delivered to the cell in a complex with a transactivator of transcription- (TAT-) protein or other cell-penetrating peptides [126]. This appears to be the most efficient solution for reprogramming without any genetic insertions (including transient transfection with plasmid DNA). In such a fashion, a culture of stable iPSCs that can sustain for over 35 passages was obtained from fibroblasts [127]. The efficiency of a protein delivery to the cell can be promoted by the implementation of chitosan nanoparticles [128] which increases the internalization of the complex by cell and transfer to the nuclear membrane. Despite the obvious attractiveness of this approach, it should be noted that the efficiency of reprogramming with help of recombinant proteins is very low, and the production of functionally active recombinant proteins is labor extensive and quite costly.

Concerning the use of mRNA or recombinant proteins for direct reprogramming, it should be noted that there is only one technology described [110], but we did not find any publications confirming the results of such a reprogramming.

## 4. Protocols of Neural Stem Cell Reprogramming

We have characterized the current protocols for the reprogramming of somatic cells into neural such as including the stage of obtaining iPSCs and the iPSC-deficient stage by direct reprogramming. Below, we present some examples of OSKM and other factors to induce NCSs and related cell types (Table 1).

### 4.1. Protocols for Obtaining NSC from iPSCs

To obtain NSC from differentiated cells, such as fibroblasts or bone marrow mononuclear cells, the investigators underline the need to develop iPSCs at the intermediate stage (Table 1). Obviously, several labs exploited the classical OTKM protocols using a viral transduction with several transcription factors for induction of reprogramming somatic cells [129, 155, 156] or separately with Oct4, Sox2, and Nanog [155]. Some groups used additional factors such as FGF-2, low concentrations of Noggin, and retinoic acid. These protocols provide an opportunity to obtain mature myelin basic protein- (MBP-) positive oligodendrocytes and bipolar GFAP-positive astrocytes [156].

For the treatment of traumatic brain injury (TBI), retroviral dual cassette vectors coexpressing the classical “OKSM” cohort in combination with eGFP were administered to the brain of TBI in the immediate vicinity of the lesion focus [129]. The expression of both human OKSM and eGFP predominantly occurs in the cells of microglia and NG2-positive oligodendrocytes (neural/glial antigen 2) and, to a much lesser extent, in GFAP-positive reactive astrocytes. The generated pool of GFP-positive cells may be considered as iPSCs due to coexpression of eGFP, Nanog, and stage-specific embryonic antigen-4 (SSEA4), with further expression of the markers of all three embryonic germ layers including ectoderm (SOX2), mesoderm (brachyury), and endoderms (Gata4), with the predominant expression of the first. Then, markers of the neuroglial lineage such as nestin and doublecortin (Dcx), as well as neuronal-specific markers NeuN and MAP2, were detected in the same cells, and even neuronal electrophysiological patterns were recorded. This technology could be proclaimed revolutionary were it not for the formation of teratomаs in the cortex of the damaged hemisphere, which were predictably the end of the experiment [129].

Other groups used predominantly nonclassical factors. For example, the team of Yaqubi et al. analyzed a combination of many and predominantly nonclassical factors for the induced conversion of murine fibroblasts to neural cells through the iPSCs stage [130]. This complex included Ezh2, Jarid2, Mtf2, Nanog, Pou5f1, Sall4, Smarca4, Sox2, Suz12, and Tcf3.

A growing body of evidence reports that the main factors of reprogramming in the neuroepithelial direction through the more or less obvious stage of iPSCs are Oct4 and Sox2 [57, 131, 138, 157]. Coexpression of these factors in cells, neural differentiation can be triggered by a change in the medium to the neuro-basal one, with the addition of appropriate supplements. In particular, the addition of N2 to the medium made it possible to obtain from the Oct4 and Sox2 positive iPSC populations of neurons and glia positive for TUJ1, GFAP, GALC, MAP2, TH, and synaptophysin [131]. Some researchers insist on the combination of Oct4 and Sox2 [157], others deny the fundamental importance of this combination and apply only one of these factors, but the latest protocols no longer contain the obvious stage of iPSCs and will be analyzed in the next section.

In general, most researchers who adhere to the “classical scheme” of reprogramming follow the general scheme of the protocol: in the first stage, the transcription factors OSKM (or all four or two of them) are used for the creation of iPSCs, in the second, for obtaining NSC and neural progenitor cells, the culture is transferred to basic neural media combined with additional factors, for example, FGF-2, EGF and Noggin and, in a number of protocols, small chemical molecules, for example VPA. In the third stage, differentiation is carried out in the neural and glial directions using neurobasic media and with the help of accompanying molecules, to which at this stage belongs forskolin, BDNF, GDNF, IGF1, NT3, and so on [110].

Recently, more and more publications have appeared, in which, in addition to or in lieu of genetic factors, the small molecules mentioned earlier were used to obtain iPSCs. To completely replace the Yamanaka factors, the following small molecules were selected by one scientific group: SB431542 (inhibitor of TGF-beta), PD0325901 (inhibitor of MEK), thiazovivin, VPA, antioxidant of ascorbic acid, and DNMT (inhibitor of 5-aza-20-deoxycytidine) [132]. They also reported that small molecules are able to suppress chromosomal aberrations at the iPSC stage. Another research team, with the aim of developing a technology for the treatment of spinal trauma, obtained iPSC-activating V2a interneurons using small molecules [133, 134]. It is hypothesized that it is this type of cells that can most effectively be incorporated into damaged afferent and efferent neuronal circuits and thus restore the damaged spinal cord.

### 4.2. Protocols for the Reprogramming of Somatic Cells in NSC that Do Not Use iPSCs

**Figure 2:**
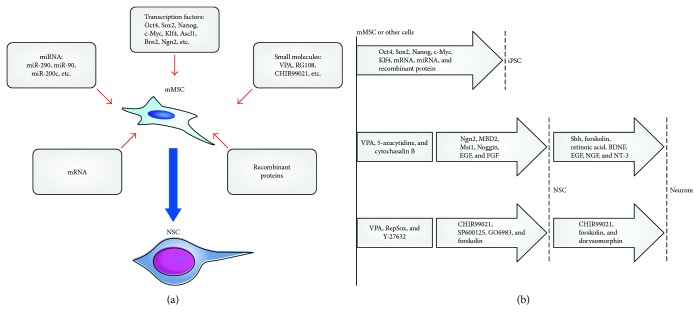
Characteristics of the main reprogramming protocols. (a) Types of transforming factors reproducing somatic cells in NSCs: transcription factors, pluripotency (Oct4, Sox2, Nanog, c-Myc, and Klf4), and factors determining proneogenic redifferentiation (Ascl1, Brn2, Ngn2, etc.); mRNA of reprogramming factors; recombinant proteins of reprogramming factors; microRNAs that help maintain pluripotency (miR-290, miR-90, miR-200c, etc.); and small chemical molecules that increase the “plasticity” of the transformed cells (VPA–histone deacetylase inhibitor (HDAC) and RG108–methyltransferase DNA inhibitor), molecules replacing by effect some transcription factors (CHIR99021 inhibitor of GSK3*β*), and so on. (b) Logical schemes of reprogramming. From top to bottom: (1) scheme in which the IPSC stage is present [94, 126, 128, 129]. (2)-(3) Schemes in which the IPSC stage is absent are divided into three main stages: preparatory (destabilizing the genome and increasing functional plasticity); the stage of redifferentiation in the NSC, and the stage of terminal neuroglial differentiation (explanations in the text). Schemes (2) and (3) extracted from [110] and [150], respectively.

In publications dedicated to the direct transdifferentiation of somatic cells into neuroepithelial ones, unlike the protocol for the production of iPSC proper, along with classical transcription factors, “nonclassical” reprogramming mixtures are much more common (Table 1, Figure 2). The principal difference of direct reprogramming is the absence or nonobviousness of the iPSC phase and the obtaining of NSC directly, by reprogramming proliferating cells, for example, fibroblasts or astrocytes. Thus, the Karen L. Ring team, using the Sox2 factor, was able to obtain Sox2-, Nestin-, Pax6-, Zbtb16-, or Msi1-positive NSC from murine fibroblasts and Sox2 and Nestin-positive NSC and MAP2-positive neurons, GFAP-positive astrocytes, and O4- and OLIG2-positive oligodendrocytes from human fibroblasts [5]. At that, additional factors were added to the cellular medium: EGF, FGF2, retinoic acid, and forskolin. In contrast to the protocols in obtaining NSC from iPSCs, no oncogenic potential was observed in the obtained neuroepithelial cells.

Another group, using direct reprogramming, obtained human motor neurons from human fibroblasts, using a combination of factors Ascl1, Brn2, Myt1l, Lhx3, Hb9, Isl1, Ngn2, and NEUROD1 [135]. As a result, induced motor neurons expressed *β*3-tubulins (Tubb2a and Tubb2b), Map2, synapsins (Syn1 and Syn2), synaptophysin (Syp), and synaptotagmins (Syt1, Syt4, Syt13, and Syt16) and had such electrophysiological characteristics of normal motor neurons as resting potential of −49.5 mV (SEM 5.6, *N* = 6), presence of active Na-K channels that were blocked by tetrodotoxin, and the ability to generate a characteristic neuronal action potential. As in the previous case, additional factors added to the medium, such as N2, B27, GDNF, BDNF, and CNTF, were used in this experiment.

Caiazzo et al. used only three transcriptional factors, NFIA, NFIB, and Sox9, to convert fibroblasts into functional astrocytes [136]. As a result, they received a stable population of functional astrocytes, positive for S100B, GLT1, ALDOC, and CD44. A group of scientists led by Cassady et al. conducted a direct reprogramming of hepatocytes and B-lymphocytes in NSC by means of a cocktail of factors Brn2, Hes1, Hes3, Klf4, Myc, Notch1, NICD, PLAGL1, and Rfx4 [137]. Cultured in a medium containing insulin, transferrin, selenin, and fibronectin (ITSFn), NSCs were positive for Nestin, and with subsequent replacement of additional EGF/FGF factors by BDNF, NT3, and ascorbic acid differentiated into TUJ1 and MAP2 positive neurons, GFAP-positive astrocytes, and O1 and O4 positive oligodendrocytes. In addition, when cells were cultivated, plates coated with polyornithine and laminin were used, which also, from the authors' point of view, contributed to the neurogenic redifferentiation of cells.

As in the case of iPSC protocols mentioned above, some researchers agree that there is no need to use an excessively large number of key factors while it may be sufficient to concentrate on one or two of the most effective ones. For example, some researchers consider one factor, Oct4 [138, 158], sufficient for reprogramming. It should be noted that CD34-positive hematopoietic cord blood cells were chosen as the original cell culture for reprogramming, which in themselves possess greater plasticity and level of stem capacity than fibroblasts and hepatocytes. As in almost all other protocols, additional factors included in the neural stem cell media are EGF, bFGF as well as Sonic hedgehog (SHH), AA platelet factor, T3 thyroid hormone, FGF-8, and GDNF at the stage of differentiation. As a result, Nestin- and Musashi-1-positive neuroglial progenitors, positive for markers of differentiated subpopulations of neurons (tyrosine hydroxylase (TH), *β*III tubulin (Tuj1), and glia) (GFAP +, and 2′,3′-cyclic nucleotide 3′-phosphodiesterase + (CNPase)), were obtained from hematopoietic cells.

Another group that used only the transcription factor Oct4 [139] for the reprogramming of human fibroblasts in NSCs also added to the cellular environment additional factors: bFGF, IGF2, N2, B27, forskolin and ascorbic acid and coated with laminin the surface of the culture plates. As a result, it was possible to form cultures of CD133-positive progenitor cells from which glia (O4 and GFAP-positive oligodendrogliocytes and astrocytes, resp.) and *β*III tubulin and MAP2 positive neurons possessing a stable neuronal action potential were obtained.

Other researchers ascribe the major role in neuroepithelial reprogramming to the Sox2 factor [57, 140, 141, 159], in some cases in combination with additional factors such as Klf4 and c-Myc, assuming, nevertheless, only Sox2 to be the leading factor [142]. Also, with Sox2 in the case of proneogenic differentiation, the factors Brn2 or Brn4 are used, resulting in neuronal progenitors and mature neurons positive for Sox2, Nestin, Msi1, CD133, N-CAM, DCX, Tuj1, and MAP2 [143–145]. Using Sox2 and FoxG1 simultaneously [145], neural progenitor cells were obtained that differentiated into Tuj1 and MAP2 positive neurons and GFAP-positive astrocytes. In these experiments, additional factors for the primary redifferentiation of fibroblasts in NSC were EGF and FGF, and the differentiation factors in mature neurons, if this problem was to be solved by the researchers, were N2, B27, retinoic acid, GDNF, and in some cases, the surface of the plates was coated with L-ornithine/laminin.

Corti et al. [146] used only individual expression of factors Oct4, Sox2, or Nanog in their work, but as the basis of successful reprogramming, the nature of the original cells is considered. As such, they used CD44+ astrocytes, reprogramming them in NSC, without the iPSCs stage, due to the greater stem potential of such astrocytes and their greater functional plasticity. As a result, the resulting NSCs were positive for Sox2, PAX6, CD133, and Nestin and subsequently were successfully differentiated into mature neurons. The same features of adult astrocytes are used by Niu et al. in their experiments [140], considering, however, sufficient exposure to them by the Sox2 and VPA factor. As a result, they obtained a population of mouse neurons positive for DCX and Sox2, which, after exposure to VPA, differentiated into more mature and functionally active neurons, positive for Sox2, calretinin, and NeuN.

In another study [141], where the researchers used only Sox2 as the main factor in the reprogramming of fibroblasts into neural stem cells, the main emphasis was placed on the three-dimensional microenvironment in which the experiment was conducted. It has been shown that such a three-dimensional microenvironment created on the basis of agarose gel, when growing cells, allows preserving or enhancing their stem potential. In addition, the researchers used auxiliary factors in the cellular environment: EGF, FGF, and B27; and for differentiation: PDGF, bFGF, forskolin, and ascorbic acid. As a result, NSCs were obtained which successfully differentiated into mature neurons, astrocytes, and oligodendrocytes, positive for Sox2, Olig2, Pax6, Tuj1, GFAP, NeuN, and Nestin. A parallel study on a two-dimensional monolayer culture showed lower results of neural differentiation. At the same time, in this work, there is a need for proneurogenic redifferentiation of fibroblasts for the addition of fetal bovine serum (FBS), without which they failed to induce reprogramming. At the same time, the overwhelming majority of the researchers mentioned above believe that the presence of fetal serum does not contribute to neuroepithelial transdifferentiation.

Some studies have been published in which one or two nonclassical transcription factors are believed to be exclusive and sufficient reprogramming factors, for example, the factors Ascl1 and Nurr1, with which it was possible to reprogram mouse fibroblasts into neural progenitor cells positive for Nestin, Sox1, and Msi1, in positive Tuj1 and MAP2 neurons, and dopaminergic neurons positively staining for TH and Tuj1 in the presence of additional factors EGF, FGF, N2, B27, ITS, and Shh [147] or oncogene SYT-SSX2 (oncogene of synovial sarcoma (SS)) [160].

Pereira et al. used three similar nonclassical factors, Ascl1, Lmx1a, and Nurr1 (ALN), to reprogram NG2 glia into various types of neurons *in vivo* and *in vitro* [148]. The resulting neurons exhibited properties of fast-spiking (FS) and parvalbumin (PV)+ interneurons (IntNs). Remarkably, the authors mention that different types of neurons may be obtained as a result of *in vivo* and *in vitro* reprogramming. In particular, by the *in vivo* reprogramming, no detection of TH positive cells that appeared *in vitro* was noted. Such difference may be due to the regional influence and heterogeneity of the epigenetic factors for glia cells *in vivo*. The heterogeneity of the expression of various factors, in the process of neural tube formation, as well as the presence of gradients for various factors, determines particularities of neuronal differentiation in ontogenesis (for a review of this, see the work of Masserdotti et al. [161]). Thus, the origin, anatomical location, and epigenetic control of reprogrammed cells may strongly affect their tendency to differentiation and transdifferentiation into certain subtypes of neurons. The same review describes in detail the role of the transcriptional factors Ascl1, Neurog1 (Ngn1), Neurog2 (Ngn2), Pax6, and others, both in the control of the embryonic development of the brain and their potential use for reprogramming. At the same time, Ascl1, Pax6, and Neurog2 are noted as the main transcription factors for proneurogenic transdifferentiation, descendingly triggering other factors.

As already noted, recently more and more studies have appeared in which small chemical molecules act as the main factors of reprogramming. For example, in the study of Zheng et al. [149], a combination of small molecules A-83-01 (inhibitor of TGF-beta), thiazovivin (ROCK inhibitor), purmorphamine (SHH agonist), and valproic acid (VPA: inhibitor of histone deacetylase (HDAC)) was chosen, resulting in the production of NSC positive for Sox2, Pax6, PLZF, Dach1, Fam70a, Nr2f1, and Zic1 from murine fibroblasts, which were then differentiated into functional neurons, astrocytes, and oligodendrocytes positive for Tuj1, GFAP, and Olig2, respectively.

In another work, a combination of six molecules was used to produce glutamatergic and GABAergic neurons from human fibroblasts: VPA, CHIR99021 (inhibitor of GSK-3 kinase), Repsox (inhibitor of TGF-*β* pathways), SP600125 (JNK inhibitor), GO6983 (PKC inhibitor), and Y-27632 (ROCK inhibitor) [150]. Another group successfully increased the activity of endogenous Sox2 to induce generation of NSC using a combination of only three chemical molecules: VPA, CHIR99021, and Repsox [113]. They also showed similar efficacy of two other combinations: the combination of NAB, LiCl, and SB431542 and the combination of TSA, Li2CO3, and tranilast. Fully functional glutamatergic neurons were obtained from astrocytes by sequential exposure to nine chemical agents: LDN193189, SB431542, TTNPB, thiazovivin, CHIR99021, VPA, DAPT, SAG, and purmorphamine [151].

In a number of other studies, small molecules are used at the stage of cell differentiation into the desired subpopulation, that is, for the production of CHX10+ V2a interneurons for the treatment of spinal trauma, the McDevitt T.C. group used purmorphamine, RA, and DAPT [133]. In all cases shown, the resulting cells had the phenotype of more or less differentiated neural cells, depending on the tasks of the particular experiment. It is worth noting that in such protocols additional factors are used, including those already mentioned above (EGF, FGF, BDNF, etc.). This indicates that they are of great importance for the stabilization of reprogramming processes, while not being sufficient for self-induction of cell redifferentiation. In general, as with other reprogramming factors, it is desirable to minimize the number of small molecules in the final combination, in order to maximize risk mitigation of negative consequences for the reprogrammed cells. To do this, research is conducted to find the most effective and universal chemical factors.

#### 4.3. General Patterns of Direct Reprogramming Protocols

Summarizing incredibly diverse protocols of direct reprogramming with obtaining neuroepithelial cells, three main stages can be distinguished (Figure 1). At the first “preparatory” stage, the main acting factors are small chemical molecules that increase the “plasticity”/destabilize the genome of the transformed cells, for example, removing de-acetylation of histones (VPA) and DNA methylation (RG108), inhibiting the TGF-*β* pathway (A-83-01), which prevents mesodermal differentiation of MMSC, and reorganizing the cytoskeleton of the cell (thiazovivin). In the second main stage, the result of which is presumably NSC, either transcription factors determining proneurogenic redifferentiation (for instance, Ascl1, Brn2, and Ngn2) or small molecules replacing these reprogramming factors are being used. For example, CHIR99021 inhibits GSK3*β* then activates the Wnt signaling pathway; BIX 01294 inhibits G9a histone methyltransferases that methylate H3K9 which presumably activates Oct4 factor, and PDO 325901 blocks the MEK signaling pathway preventing differentiation. In both cases, additional factors EGF, FGF, and so on are also used at the main stage. In the third and final stage, differentiation into the desired cell type is carried out, for example, in neurons using small chemical molecules (purmorphamine (SHH agonist), retinoic acid, and forskolin (AMP activator)), and additional factors of neural differentiation, that is, BDNF and GDNF.

## 5. Advantages and Disadvantages of Reprogramming Approaches

### 5.1. iPSCs Technology

The first obstacle to clinical using iPSCs is the lack of effective and complete differentiation of immature iPSCs to mature somatic cells. The potential tumorigenicity of pluripotent factors and the preferential use of retroviral and lentiviral transduction may lead to the development of insertional mutagenesis, activation of unwanted gene expression, genomic instability, and chromosomal aberrations [162]. In addition, although autologous iPSCs are considered nonimmunogenic, they can induce an immune response after transplantation by inducing genetic and epigenetic instability [163].

Despite the original attractiveness, clinical use of iPSCs can presumably represent a risk. Approaches of delivery along with the nature of the pluripotent factors and each stage of the construction of pluripotency of cells should be strictly controlled to escape skewing to the malignant transformation.

### 5.2. Direct Reprogramming

The advantages of direct reprogramming are obvious: firstly, this technology is much faster and cheaper than iPSCs, and, arguably, from the standpoint of economic feasibility, provides a realistic possibility for obtaining autologous neural stem cells, at least until the banks of iPSC haplotypes are fully formed. Secondly, this technology appears to be safer than iPSCs, because even by skipping the stage of integrating the viral genome into the DNA of the reprogrammed cells, activation of oncogenic pluripotency factors may potentially contribute to tumor formation.

As a disadvantage of direct reprogramming, its reduced predictability may be mentioned; since, in this case, it is possible to simultaneously obtain cells from various stages of differentiation, that is, NSC, NPK, late neural and glial progenitors, and terminally differentiated cells. The very same specific feature of direct reprogramming protocols shows that their reproducibility and sensitivity to the conditions of the experiment are decidedly inferior to those of the iPSC protocols.

## 6. Conclusions

Uniqueness of stem cells and their potential for use in scientific and therapeutic applications is based on their ability to self-renewal and pluripotency. The state of pluripotency, natural and effectively controlled in ESC, is formed due to signaling pathways as well as transcription and other epigenetic factors. It is already evident that all these factors form a complex self-regulating multilevel system. Assuredly, the possibilities of acquiring and maintaining pluripotency are fundamentally different in ESC and adult somatic cells. Unlike ESC, the activation of prooncogenic pluripotent factors in the framework of the somatic cells reprogramming almost always leads to the development of teratomas because of the insufficient molecular control over the signaling pathways. Thus, a serious deficiency in reprogramming factors and methods of their delivery into the cell forces the researchers to look for ways to optimize their reprogramming protocols and reduce the number of aggressive influences. Problems associated with the unstable state of iPSCs initiated the development of alternative technologies for direct reprogramming of somatic cells, circumventing the dangerous stage of poorly controlled activation of prooncogens of OSKM. Another reason for the need to develop a successful strategy of direct reprogramming is the economic benefit. Because of their high scost, iPSCs are extremely expensive therapy, especially within the framework of personalized medicine. An alternative strategy of direct reprogramming allows less effort to obtain multipotent, progenitor, and differentiated cells to solve specific clinical and scientific problems with a much lower risk of tumorigenicity, due to their lessened genetic instability. However, the problem of the lack of molecular control of differentiation in direct reprogramming has not yet been solved, and, therefore, there are no generally accepted effective and reproducible methods for direct reprogramming of somatic cells in the NSC.

## Figures and Tables

**Table 1:** Currently existing protocols for reprogramming/transdifferentiation.

Primary cell type	Factors used	Resulting cell type	Reference
Mouse glial cells *in vivo*	Retroviral transduction of Oct4, Klf4, Sox2, and c-Myc (OKSM)	iPSCs	[129]
Mouse fibroblasts	Retroviral transduction of Oct4, Klf4, Sox2, and transfection miR-294	ESC	[93]
Mouse adipose stromal cells (mASCs), human adipose stromal cells (hASC), and human dermal fibroblasts (HDFs)	Transfection with miR-200c, miR-302, and miR-369	iPSCs	[94]
Mouse fibroblasts	Lentiviral transduction by miR-302 and miR-367	iPSCs	[95]
Mouse fibroblasts	Bioinformatic analysis on microarrays Ezh2, Jarid2, Mtf2, Nanog, Pou5f1, Sall4, Smarca4, Sox2, Suz12, and Tcf3	NSC	[130]
iPSCs from embryonic NSC	Retroviral transduction of Oct4 and Sox2	Neurons	[131]
Mouse fibroblasts	SB431542, PD0325901, thiazovivin, VPA, and antioxidant of ascorbic acid and DNMT	iPSCs	[132]
iPSCs and ESC	Purmorphamine, RA, and DAPT	V2a interneurons	[133, 134]
Mouse fibroblasts and human fibroblasts	Retroviral transduction Sox2	NSC	[5]
Human fibroblasts	Retroviral transduction with Ascl1, Brn2, Myt1l, Lhx3, Hb9, Isl1, Ngn2, and NEUROD1	Motor neurons	[135]
Mouse fibroblasts	Lentiviral transduction with NFIA, NFIB, and Sox9	Astrocytes	[136]
Hepatocytes and B-lymphocytes	Lentiviral transduction with Brn2, Hes1, Hes3, Klf4, Myc, Notch1, NICD, PLAGL1, and Rfx4	NSC	[137]
CD34-positive cells of umbilical cord blood	Lentiviral transduction with Oct4 and transfection with a plasmid containing Oct4	NSC	[138]
Human fibroblasts	Lentiviral transduction with Oct4	Neural progenitor cells	[139]
CD44+ аstrocytes	Lentiviral transduction with Sox2 and VPA	Neurons	[140]
Mouse fibroblasts	Lentiviral transduction with Sox2	Neural progenitor cells	[141]
Mouse fibroblasts	Retroviral transduction with Sox2, Klf4, and c-Myc	NSC	[142]
Human fibroblasts	Lentiviral transduction with Sox2, с-Мус, and Brn2/Brn4	Neurons	[143]
Human fibroblasts	Retroviral transduction with Sox2, с-Мус, and Brn4	NSC	[144]
Mouse fibroblasts	Lentiviral transduction with Sox2 and FoxG1	Neural progenitor cells	[145]
CD44+ аstrocytes	Lentiviral transduction with Oct4/Sox2/Nanog	NSC	[146]
Mouse fibroblasts	Retroviral transduction with Ascl1, Nurr1, and Shh	Neural progenitor cells, neurons and dopaminergic neurons	[147]
Mouse NG2-glia *in vivo* and *in vitro*	AAV5 transduction with Ascl1, Lmx1a, and Nurr1 (ALN)	Neurons having properties of fast-spiking (FS) and parvalbumin (PV)+ interneurons (IntNs)	[148]
Mouse fibroblasts	А-83-01, thiazovivin, purmorphamine, and VPA	NSC	[149]
Human fibroblasts	VPA, CHIR99021, Repsox, SP600125, GO6983, and Y-27632	Glutamatergic and GABAergic neurons	[150]
Mouse fibroblasts	CHIR99021, VPA, Repsox, LiCl and SB431542/TSA, Li2CO3 and tranilast	NSC	[113]
Human astrocytes	LDN193189, SB431542, TTNPB, thiazovivin, CHIR99021,VPA, DAPT, SAG, and purmorphamine	Glutamatergic neurons	[151]
Mouse astrocytes	Retroviral transduction by Ascl1 + Dlx2, Neurog2, and Dlx2	Glutamatergic and GABAergic neurons	[152]
Mouse embryonic and postnatal fibroblasts	Lentiviral transduction by Ascl1, Bmp2, and Myt1l	Glutamatergic and GABAergic neurons	[153]
Human fibroblasts	Lentiviral transduction with miR-124, Bmp2, Myt1l, Noggin, and FK	Glutamatergic and GABAergic neurons	[154]
